# The Rad9–Rad1–Hus1 DNA Repair Clamp is Found in Microsporidia

**DOI:** 10.1093/gbe/evac053

**Published:** 2022-04-19

**Authors:** Anne Caroline Mascarenhas dos Santos, Alexander Thomas Julian, Jean-François Pombert

**Affiliations:** Department of Biology, Illinois Institute of Technology, Chicago, IL, USA; Department of Biology, Illinois Institute of Technology, Chicago, IL, USA; Department of Biology, Illinois Institute of Technology, Chicago, IL, USA

**Keywords:** *Encephalitozoon*, DNA damage response, computational biology, genome annotation, protein structure prediction, structural homology

## Abstract

DNA repair is an important component of genome integrity and organisms with reduced repair capabilities tend to accumulate mutations at elevated rates. Microsporidia are intracellular parasites exhibiting high levels of genetic divergence postulated to originate from the lack of several proteins, including the heterotrimeric Rad9–Rad1–Hus1 DNA repair clamp. Microsporidian species from the Encephalitozoonidae have undergone severe streamlining with small genomes coding for about 2,000 proteins. The highly divergent sequences found in Microsporidia render functional inferences difficult such that roughly half of these 2,000 proteins have no known function. Using a structural homology-based annotation approach combining protein structure prediction and tridimensional similarity searches, we found that the Rad9–Rad1–Hus1 DNA clamp is present in Microsporidia, together with many other components of the DNA repair machinery previously thought to be missing from these organisms. Altogether, our results indicate that the DNA repair machinery is present and likely functional in Microsporidia.

**Significance**

Microsporidia are obligate intracellular pathogens with poorly understood proteomes stemming from high levels of genetic diversity that befuddle traditional sequence-based functional inference methods. This genetic diversity was postulated to originate from large gaps in the eukaryotic DNA repair machinery but here we showed that this is not the case. Using genome-wide searches leveraging the latest tools in structural homology, we showed that Microsporidia code for a much more complete DNA repair proteome than previously thought, thus challenging our previous hypotheses about why these organisms are so divergent at the sequence level.

## Introduction

Genome maintenance and integrity require DNA replication and repair processes (Choi and Chung 2020). Organisms that lack DNA repair mechanisms tend to accumulate mutations at elevated rates, but pathogenic organisms such as viruses and parasites can benefit from faster mutation rates that quicken the pace of their adaptation against host defenses (Siao et al. 2020). Microsporidia is a diverse and successful fungal-related lineage of obligate intracellular parasites that infect a wide range of hosts, and whose diversity is reflected at the genetic level (Pombert et al. 2013; Wadi and Reinke 2020). Microsporidian genomes not only exhibit remarkably high levels of sequence divergence (Pombert et al. 2013) but also differ in size by as much as an order of magnitude, from <3 Mbp in human-infecting *Encephalitozoon* spp. (Corradi 2015) to more than 50 Mbp in the mosquito parasite *Edhazardia aedis* (Desjardins et al. 2015). Albeit microsporidians constitute excellent models to study the evolution of parasitism from a genomic perspective (Wadi and Reinke 2020), their high levels of sequence divergence render functional inferences difficult. As such, about half of their proteome has yet to be assigned any function (Pombert et al. 2015), which greatly limits our understanding of what these organisms are truly capable of.

The high levels of sequence divergence observed across microsporidia lineages were postulated to originate from the lack of several common eukaryotic DNA repair proteins (Corradi 2015; Galindo et al. 2018), including Rad9–Rad1–Hus1, Rad17, and DDB1–Cul4 (Gill and Fast 2007). The Rad9–Rad1–Hus1 checkpoint clamp, better known as the 9–1–1 complex, is a heterotrimeric ring composed of the proteins Rad9, Rad1, and Hus1 (Ddc1, Rad17, and Mec3 in yeast) and forms a structural analog of the well-known proliferating cell nuclear antigen (PCNA) homotrimeric DNA clamp (Bermudez et al. 2003; Doré et al. 2009; Sohn and Cho 2009). The 9–1–1 complex binds more tightly than PCNA to DNA (Querol-Audí et al. 2012), and acts as a scaffold in several DNA repair mechanisms including homologous recombination (HR) (Sun et al. 2020), base excision repair (Luncsford et al. 2010), and nucleotide excision repair (NER) (Li et al. 2013). The 9–1–1 complex is also involved in the maintenance of telomeres (Francia et al. 2006) and plays a balancing act between genome stability and plasticity in the human-infecting protozoan *Leishmania* (Damasceno et al. 2018).

Unfortunately, many unicellular organisms like *Encephalitozoon* spp. exhibit very high levels of divergence at the sequence level, which severely impacts our ability to predict the function of their proteins by traditional approaches based on sequence homology. However, because shape often confers function in biology, we can also look at the tridimensional (3D) shapes of proteins to try to infer their function by structural homology. Predicting the function of proteins by structural homology-based approaches requires their 3D structures, which are queried against other 3D structures for potential matches, but because the process of solving 3D structures experimentally is onerous and time consuming, only a few *Encephalitozoon* proteins are available in the RCSB Protein Data Bank (PDB) (Burley et al. 2021). This gap in experimental knowledge can be filled by computational predictions. Although traditionally shunned due to their heavy computational requirements and limited accuracy, predictive methods have made great strides in the last decade (Kuhlman and Bradley 2019)—best exemplified by the transformative results achieved by the AlphaFold2 team in the CASP14 competition (Callaway 2020)—and predicted structures are now often good enough to act as substitutes for structural homology purposes. This approach has been used to help annotate the proteins from the parasitic protist *Giardia* (Ansell et al. 2019), and recently we developed a pipeline titled 3DFI to help infer protein function from genome-wide structural homology searches (Julian et al. 2021).

In this manuscript, to account for the high levels of sequence divergence in microsporidia and better understand their resilience to both endogenous and exogenous types of DNA damage, we leveraged genome-wide structural homology-based approaches to reinvestigate the *Encephalitozoon cuniculi* GB-M1 proteome and help identify many of its previously missing DNA repair components.

## Results

### The Rad9–Rad1–Hus1 Clamp and Associated Components are Found in Microsporidia

**Fig. 1.**
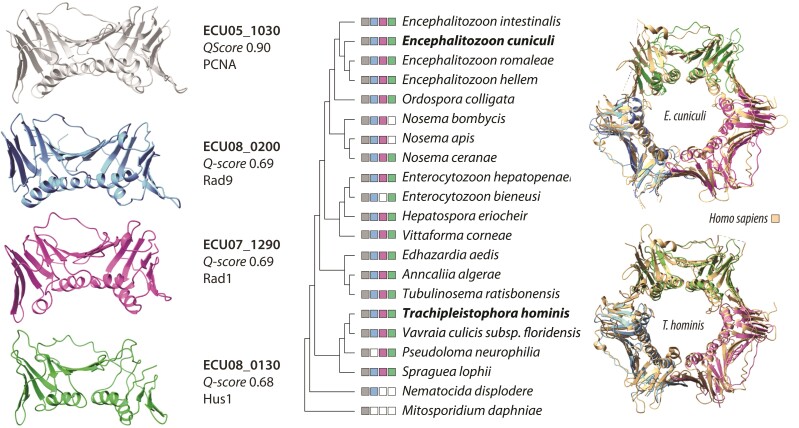
Distribution of the Rad9–Rad1–Hus1 DNA repair clamp in Microsporidia. (*Left*) RaptorX predicted 3D structures of *E. cuniculi* PCNA-like proteins identified with GESAMT. (*Center*) Distribution of PCNA-like proteins in microsporidia as inferred by PSI-BLAST searches. (*Right*) ChimeraX overlap of the *E. cuniculi*/*T. hominis* Rad9–Rad1–Hus1 structural homologs against the human 9–1–1 crystal structure (3A1J; Sohn and Cho 2009).

The 9–1–1 complex is a heterotrimer composed of the Rad9, Rad1, and Hus1 proteins and is structurally analogous to the PCNA homotrimeric DNA clamp (Bermudez et al. 2003; Doré et al. 2009). A total of four PCNA-like proteins with structural alignment scores (*Q*-score) ≥ 0.68 against PCNA (fig. 1, left panel) were found encoded in the *E. cuniculi* genome (supplementary table S1 and figs. S1 and S2, Supplementary Material online). These included the already known PCNA (ECU05_1030) and three proteins (ECU07_1290, ECU08_0130, ECU08_0200) of previously unknown functions (Gill and Fast 2007; Pombert et al. 2013). Gene ontology searches performed in the 3D space further supported the involvement of these proteins in DNA repair processes (supplementary data S1, Supplementary Material online). When we overlapped the predicted structures of these three proteins against the crystal structure of the human 9–1–1 complex (Sohn and Cho 2009), they each aligned well with one of the Rad9, Rad1, and Hus1 subunits (fig. 1, right panel). Quality assessment of the predicted structures performed independently with the VoroCNN deep convolutional neural network (Igashov et al. 2021) indicated that these structures were accurately folded (supplementary fig. S3, Supplementary Material online), and round-robin comparisons between the per-protein AlphaFold models (models 1–5) revealed very similar structures between the different models (supplementary table S2, Supplementary Material online). Further reconstruction of the PCNA and 9–1–1 protein complexes with AlphaFold-Multimer (Evans et al. 2021) properly recreated the homo- and heterotrimer structures of these complexes (supplementary fig. S4, Supplementary Material online).

**Fig. 2.**
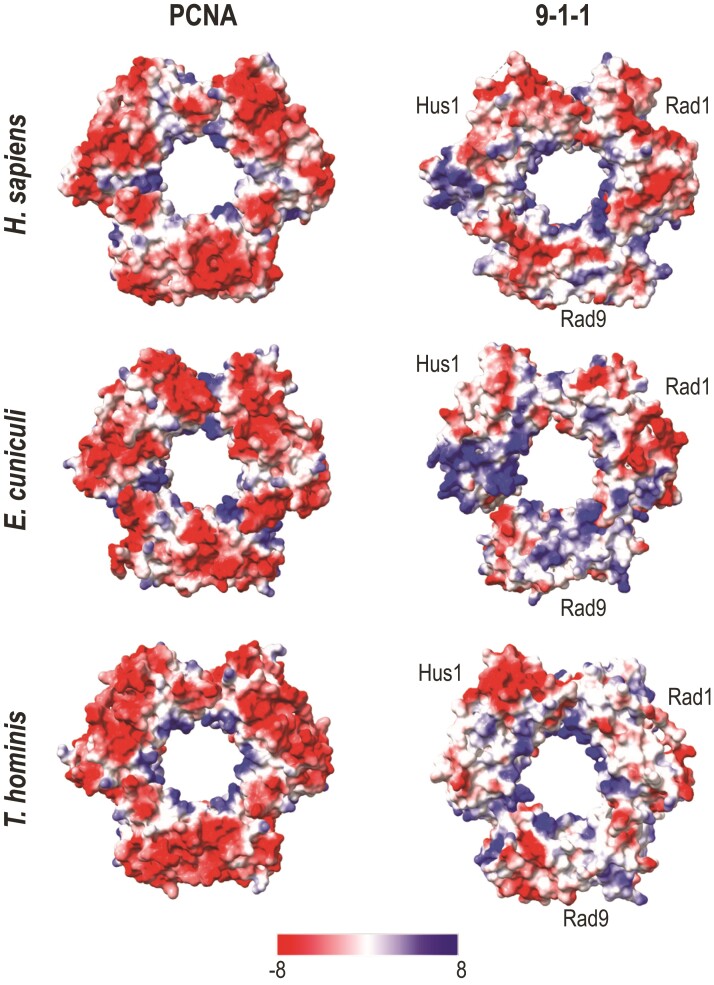
Electrostatic potentials of the PCNA and Rad9–Rad1–Hus1 complexes. Human experimental structures were downloaded from RCSB PDB (accession numbers 3JA9 [PCNA; Lau et al. 2015] and 3AIJ [9–1–1; Sohn and Cho 2009]). *Encephalitozoon cuniculi* and *T. hominis* structures were predicted with RaptorX. The electrostatic potential values range from −8 kcal/mol·*e* to +8 kcal/mol·*e*.

To verify that the 9–1–1 complex is not exclusive to *E. cuniculi*, we searched for the presence of PCNA, Rad9, Rad1, and Hus1 across several representative microsporidia species (fig. 1, center panel). The PCNA, Rad9, Rad1, and Hus1 subunits were found in most microsporidia, including the distant *Trachipleistophora hominis*, and to ensure that these orthologs inferred by PSI-BLAST searches were real and not spurious hits, we applied the same 3D approach to orthologs detected in *T. hominis*. The *T. hominis* PCNA (THOM_2122; Q-score 0.88), Rad9 (THOM_2652; Q-score 0.69), Rad1 (THOM_2045; Q-score 0.78), and Hus1 (THOM_0248; *Q*-score 0.54) orthologs yielded similar structures to the reference, as expected. Overall amino acid identity/similarity between the *E. cuniculi* and *T. hominis* PCNA-like structures and their distant human counterparts averaged 29.42%/69.48% (PCNA), 9.84%/52.19%(Rad9), 11.14%/47.62% (Rad1), and 12.31%/55.04% (Hus1) and, despite low identity (supplementary fig. S5, Supplementary Material online), predicted electrostatic potentials were found congruent with the expected differences between the PCNA and 9–1–1 DNA clamps (fig. 2) (Sohn and Cho 2009).

The 9–1–1 complex, like the PCNA clamp, is unable to load itself onto DNA and requires a clamp loader to be properly mounted at sites of DNA damage (Acevedo et al. 2016). The PCNA clamp loader is composed of five replication factor C (RFC) subunits (1–5), and the 9–1–1 complex utilizes the same proteins with the exception of RFC1, which is replaced by Rad17 in humans or Rad24p in yeast (Bermudez et al. 2003; Doré et al. 2009). The *E. cuniculi* genome was found to encode a total of six RFC-like subunits (table 1), consistent with the presence of the two DNA clamps. Using structural homology, we were able to assign these subunits to their specific yeast counterparts and to differentiate between the microsporidian RFC1 (ECU05_1530) and Rad17 (ECU01_1180), the latter corroborated by PFAM motifs searches. Recruitment of the 9–1–1 complex also requires the presence of DNA topoisomerase topBP1 (Acevedo et al. 2016), previously lacking from *E. cuniculi* genome annotations (Pombert et al. 2013), and using PSI-BLAST searches with the human topBP1 as query we identified this protein as ECU02_1320, a result corroborated by 3D folding and structural similarity searches (table 1). 9–1–1 loading is further facilitated by replication protein A (RPA), previously identified in microsporidia (Gill and Fast 2007; Yan and Michael 2009). RPA is a heterotrimer composed of subunits RPA1, RPA2, and RPA3 that bind and coat single-stranded DNA. Interactions between the RPA-coated DNA, DNA-mounted 9–1–1 complex, and topBP1 are primordial for the activation of the checkpoint signaling cascade (Acevedo et al. 2016). This activation requires the ataxia telangiectasia-mutated and Rad3-related (ATR)/ATR-interacting protein (ATRIP) regulator of DNA damage response (Mec1/Ddc2 in yeast), and ATR was identified in *E. cuniculi* as ECU02_1130 but the presence of ATRIP could not be ascertained by structural homology.

**Table 1:** Rad9–Rad1–Hus1-Related Proteins Found in *E. cuniculi*

		Inference Method					
Locus Tag	Product	Pfam	PSI-BLAST	3D	PDB Reference^a^	RMSD^b^	Average Expression^c^
ECU05_1030	PCNA	+	+	+	6E49	0.703	850.96
ECU08_0200	Rad9	−	−	+	3A1J	1.287	182.42
ECU07_1290	Rad1	−	−	+	3A1J	1.298	216.06
ECU08_0130	Hus1	−	−	+	3A1J	1.147	81.56
ECU01_1180	Rad17	+	+	+	1SXJ	1.059	61.08
ECU05_1530	RFC1	+	+	+	1SXJ	0.660	224.28
ECU02_0290	RFC2	+	+	+	1SXJ	1.014	345.23
ECU02_0680	RFC3	−	+	+	1SXJ	0.000	289.88
ECU09_1330	RFC4	+	+	+	1SXJ	1.153	260.70
ECU10_0780	RFC5	−	+	+	1SXJ	0.001	229.67
ECU06_0360	RPA2	−	+	+	1L1O	0.805	710.66
ECU07_0950	RPA3	+	+	+	1L1O	0.836	574.63
ECU10_0600	RPA1	+	+	+	1L1O	0.956	667.47
ECU02_1320	TopBP1	−	+	+	3AL2	0.718	89.07
ECU02_1130	ATR	−	+	+	5YZ0	1.066	116.82

a Yeast and human PDB reference structures used for manual comparisons with ChimeraX; yeast structures (6E49 and 1SXJ), human structures (3A1J, 1L1O, 3AL2, and 5YZ0).

b Root mean square deviations (RMSD) of compared 3D structures in angstroms (pruned pairs) calculated with ChimeraX.

c Average expression levels inferred from RNA data by Grisdale et al. (2013).

To check if the DNA damage checkpoint pathway is active in Encephalitozoonidae, we used the available *E. cuniculi* transcriptomic data (Grisdale et al. 2013) to assess the expression levels of the corresponding genes (table 1). All genes were found expressed in *E. cuniculi*, with the PCNA subunit expressed at greater levels than the PCNA-like Rad9, Rad1, and Hus1 subunits, consistent with the homotrimeric and heterotrimeric nature of the PCNA and 9–1–1 clamps, respectively. Altogether, the presence of the Rad9–Rad1–Hus1, RFC2–5, Rad17, topBP1, ATR, and RPA1–3 proteins and their expression levels indicate that this pathway is functional in Encephalitozoonidae.

### The Cul4–DDB1 Complex is Also Found in Microsporidia

The Cul4–DDB1 complex is part of the NER and its two subpathways: the transcription-coupled (TC) NER and global-genome (GG) NER (Chalissery et al. 2017). The two differ in how they recognize helix-distorting DNA lesions but otherwise share DNA damage verification, lesion excision, synthesis, and ligation steps (Chalissery et al. 2017). Although most of the proteins involved in the later stages have been found in microsporidia, many of the proteins involved in DNA lesion recognition have yet to be identified (Gill and Fast 2007; Kanehisa et al. 2021).

**Fig. 3.**
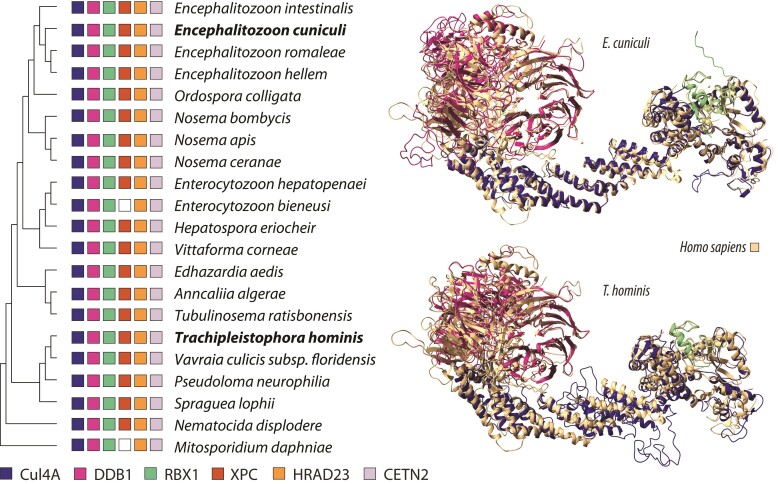
Distribution of the Cul4–DDB1–RBX1 and XPC–HRAD23–CETN2 complexes in Microsporidia. (*Left*) Presence/absence of the NER lesion recognition proteins across representative microsporidia species as inferred by PSI-BLAST searches. (*Right*) ChimeraX alignments of the *E. cuniculi*/*T. hominis* Cul4–DDB1–RBX1 structural homologs predicted with RaptorX and SWISS-MODEL, respectively, against the human crystal structure (2HYE; Angers et al. 2006).

The TC-NER subpathway recognizes lesions on DNA strands being actively transcribed and is triggered by RNA polymerase II stalling (Wang 2020). This pathway requires the Cul4, DDB1, and RBX1 proteins (Rtt101, Mms1, and Hrt1 in yeast, respectively) to sense UV-induced cyclobutene pyrimidine dimers (CPDs) together with CSA and CSB (Rad28 and Rad26 in yeast, respectively) (Chalissery et al. 2017; Wang et al. 2018; Wang 2020). The Cul4, DDB1, and CSA proteins were lacking from microsporidian genome annotations, but we were able to identify two copies of Cul4 (ECU06_0880 and ECU09_1810) and three of DDB1 (ECU05_1150, ECU07_1670, and ECU11_0610) in *E. cuniculi* using structural homology searches, with AlphaFold-multimer reconstructions of the DDB1–Cul4–RBX1 protein complexes producing the expected structures (fig. 3; supplementary fig. S6, Supplementary Material online). Unfortunately, however, the presence of CSA in Microsporidia could not be ascertained due to its seven-bladed single β-propeller structure, a repetitive fold commonly found in many proteins (Henning et al. 1995; Schapira et al. 2017) (supplementary table S3, Supplementary Material online).

The GG-NER subpathway detects DNA lesions genome-wide using the UV-damage DNA-binding (UV-DDB) and the broad specificity XPC–HRAD23–CETN2 (Rad4–Rad23–Rad33 in yeast) protein complexes (Kusakabe et al. 2019). The UV-DDB complex is a heterodimer composed of DDB1 (Mms1 in yeast) and DDB2 that can also form a larger complex with the Cul4–RBX1 ubiquitin ligase (Rtt101–Hrt1 in yeast) to promote the downstream activation of NER following recognition of UV photolesions (Kusakabe et al. 2019), whereas the XPC–HRAD23–CETN2 complex recruits the versatile transcription initiation factor TFIIH complex to promote unwinding and the opening of the DNA helix (Compe and Egly 2012). Although Cul4, RBX1, and DDB1 are found in *E. cuniculi* (table 2), the presence of DDB2—another seven-bladed single β-propeller structure (Fischer et al. 2011)—could not be ascertained by structural homology (supplementary table S3, Supplementary Material online). However, because the budding yeast uses a DDB2-independent complex composed of Rad7–Rad16 to repair CPDs (Liu et al. 2019), we also searched for Rad7 and Rad16 homologs in *E. cuniculi* using both sequence and structural homology searches. Unfortunately, no Rad7 nor Rad16 homolog could be identified. In contrast, structural homologs of XPC (ECU01_0450), HRAD23 (ECU07_0290; putative), and CENT2 (ECU03_1570 and ECU09_1220) were found in *E. cuniculi* (table 2). TFIIH subunits TTDA (ECU09_1615), CDK7 (ECU02_1450) and MNAT1 (ECU11_0220), together with two additional XPD copies (ECU08_1120 and ECU02_1090) were further identified by structural homology (table 2).

**Table 2:** TC-NER and GG-NER Proteins Identified in *E. cuniculi*

		Inference Method					
Locus Tag	Product	Pfam	PSI-BLAST	3D	PDB Reference^a^	RMSD^b^	Average Expression^c^
ECU06_0880	Cul4	−	−	+	2HYE	1.623	224.52
ECU09_1810	Cul4	−	+	+	2HYE	3.030	139.69
ECU05_1150	DDB1	−	−	+	2HYE	1.025	111.70
ECU07_1670	DDB1	−	−	+	2HYE	1.000	123.30
ECU11_0610	DDB1	−	−	+	2HYE	1.522	201.62
ECU01_1095	RBX1	+	−	+	1U6G	0.734	304.60
–	CSA	−	−	−	6FCV	–	–
ECU09_0410	CSB	−	+	+	5VVR	1.145	204.75
–	DDB2	−	−	−	4A0A	–	–
ECU01_0450	XPC	+	+	+	4YIR	1.051	96.41
ECU07_0290	HRAD23	−	−	+	1OQY	0.852	2977.13
ECU03_1570	CETN2	−	−	+	2GGM	2.188	2209.99
ECU09_1220	CETN2	−	−	+	2GGM	2.124	1366.88
–	TFIIH1	−	−	−	6NMI	–	–
ECU09_1615	TTDA	+	+	+	6NMI	1.244	69.55^d^
–	CCNH	−	−	−	1JKW	–	–
ECU06_0200	XPD	+	−	+	6NMI	1.352	153.51
ECU02_1090	XPD	+	−	+	6NMI	1.312	124.19
ECU08_1120	XPD	−	−	+	6NMI	2.533	104.46
ECU02_1450	CDK7	−	−	+	1UA2	0.619	124.62
ECU11_0220	MNAT1	+	+	−	6NMI	–	209.29

note.—(–) Proteins that could not be identified in microsporidia by structural homology.

a Yeast and human PDB reference structures used for manual comparisons with ChimeraX; yeast structures (5VVR and 4YIR), human structures (2HYE, 1U6G, 6FCV, 4A0A, 1OQY, 2GGM, 6NMI, 1JKW, and 1UA2).

b Root mean square deviations (RMSD) of compared 3D structures in angstroms (pruned pairs) calculated with ChimeraX.

c Average expression levels inferred from RNAseq data by Grisdale et al. (2013).

d Gene missing from the of *E. cuniculi* GB-M1 NCBI annotation (accession GCF_000091225.1); added manually before calculation.

All TC-NER and GG-NER genes identified in this study were found to be expressed in *E. cuniculi* (table 2), and homologs of Cul4, DDB1, RBX1, CSB, XPC, HRAD23, and CETN2 were found across representative microsporidian species (fig. 3, left panel). Again, to ensure that these were not spurious hits, the *T. hominis* homologs identified with PSI-BLAST (DDB1: THOM_0565, THOM_1591; Cul4: THOM_0276) and hidden Markov model (HMM) searches (RBX1: THOM_2073) were folded and aligned against reference structures (fig. 3, right panel).

### Other DNA Repair Pathways Components

We also investigated the *E. cuniculi* predicted proteome for a few select proteins that were missing from its otherwise mostly complete base excision repair and HR pathways (Gill and Fast 2007). The base excision repair (BER) pathway detects nonbulky DNA damage usually caused by oxidation or deamination of nitrogenous bases (Chalissery et al. 2017; Beard et al. 2019). BER DNA lesion recognition relies on the activity of specialized glycosylases, for example, the 8-oxoguanine-DNA N-glycosylase (OGG1; ECU08_0770 in *E. cuniculi* [Gill and Fast 2007]), which senses guanines oxidized to 8-dihydro-7,8-oxoguanosine (8-oxodG) and removes them from DNA before downstream replication processes (Chalissery et al. 2017). Using a combination of structural homology and PSI-BLAST searches, we identified MUTYH (MutY homolog) as ECU08_0880 in *E. cuniculi*, a DNA glycosylase that removes adenines improperly paired to 8-oxodG (Russelburg et al. 2020). The HR pathway is an error-free DNA repair mechanism active in the S and G2 phases of the cell cycle that repairs double-stranded breaks using the sister chromatid DNA strand as a template (Sun et al. 2020), and whose components are known to interact with the ataxia-telangiectasia mutated kinase (Zhou et al. 2020). During HR, strand invasion and nucleosome mobilization steps are mediated with the help of Rad54 (Zhou et al. 2020), now identified as ECU09_0410 (*Q*-score 0.52) in *E. cuniculi*.

In contrast, structural homology searches for missing components of the mismatch repair (MMR) pathway proved unsuccessful. MMR recognizes and corrects improperly matched DNA bases and insertions/deletions (indels) during replication, repair, and recombination processes with the help of the MutSα or MutSβ complexes, respectively (Liu et al. 2017). MutSα is a heterodimer composed of MSH2 and MSH6, whereas in MutSβ, MSH6 is replaced by the structural analog MSH3 (Pal et al. 2020). MSH2 and MSH6 were previously identified by sequence homology searches in *E. cuniculi* as ECU03_0540 and ECU10_0710, respectively, but no homolog of MSH3 has been identified yet. Structural homology searches confirmed the presence of MSH2 (ECU03_0540; *Q*-score 0.6 against RCSB PDB structure 2O8B chain A) but retrieved only a single MSH6/MSH3-like analog (ECU10_0710; *Q*-score of 0.5 against 3THZ chain B; see supplementary data S5, Supplementary Material online), suggesting that MSH3 might indeed be missing from *E. cuniculi*.

## Discussion

Identifying the functions of predicted proteins is an important step in deciphering the genetic blueprint of any organism, and in silico inference methods are often employed to help tackle the massive amount of data generated by genome sequencing projects. However, because traditional in silico inference methods based on sequence homology can fail when in presence of highly divergent sequences and/or understudied organisms, many proteins remain annotated as hypothetical in genome projects. When we began this study, we aimed to identify many of the unknown proteins found in NIAID Category B human pathogens from the genus *Encephalitozoon* by using the latest advances in structural homology. At the time, only template-based predictive methods were available, but these were sufficient to identify the presence of four PCNA-like structural analogs in *E. cuniculi*, which led us to rethink what we really know about DNA repair in microsporidia. Pathogens are locked in an ever-evolving molecular warfare with their hosts, with high mutation rates fastening the pace of adaptation to their host defenses, and the high levels of sequence divergence found in microsporidian species were hypothesized to originate from gaps in their DNA repair capabilities (Corradi 2015; Galindo et al. 2018), but is that really the case?

Pathogens often discard components that they no longer need upon conversion to an obligate intracellular parasitic lifestyle, and microsporidia from the genus *Encephalitozoon* are paragons of streamlining (Pombert et al. 2012) with eukaryotic genomes clocking in at <3 Mbp and encoding a mere 2,000 or so proteins. With such a thorough pruning of molecular functions, one can intuit that the proteins that remain have been kept because they are needed. Which begs the question, why keep the 9–1–1 SOS DNA repair ring, its accessory components, and the DDB1–Cul4–RBX1 and XPC–HRAD23–CETN2 DNA lesion recognition complexes if not to use them? The presence of these DNA repair complexes in *E. cuniculi* and across microsporidia (figs. 1 and 3) does indeed suggest that these organisms are more resilient to DNA damage than originally thought. Using available *E. cuniculi* RNAseq data (Grisdale et al. 2013), we confirmed that key DNA damage response genes are expressed in *E. cuniculi* GB-M1 (table 1), further indicating that these genes are likely functional and not just remnants that have yet to be streamlined out of the *Encephalitozoon* genetic paraphernalia. However, although there is no doubt that the microsporidian DNA repair proteome is larger than previously anticipated, there is no guarantee that the corresponding proteins are as effective at repairing DNA as those from other eukaryotes.

In microbial organisms, hypermutable isolates (also known as hypermutators) often arise from mutations in DNA repair components, notably genes involved in MMR (Rees et al. 2019), and several human-infecting lineages of fungi—to which Microsporidia are closely related (Choi and Kim 2017)—adapt to their host defenses and develop resistance to drugs by relying on hypermutator phenotypes (Boyce et al. 2017). In the fungal pathogens *Cryptococcus neoformans* (Boyce et al. 2017) and *Candida glabrata* (Healey et al. 2016), hypermutator phenotypes caused by mutations in the MMR protein MSH2 were associated with high genome variability and drug resistance (Boyce et al. 2017; Beekman and Ene 2020) and, in the nonpathogenic yeast *Saccharomyces cerevisiae*, defects in the MSH6/MSH3 structural analogs have been associated with hypermutable isolates (Harrington and Kolodner 2007). The presence of MSH6 but the apparent absence of MSH3 from the *E. cuniculi* DNA repair proteome combined with the overall high levels of sequence divergence observed for its identified components (many of which could only be identified by structural homology) suggests that *Encephalitozoon* species might also leverage similar mechanisms to achieve hypermutability. Other mechanisms associated with high mutation rates in pathogenic fungi include noncanonical DNA damage responses (Shor et al. 2020) and ploidy changes/loss-of-heterozygosity (LOH) (Beekman and Ene 2020), but we did not observe any evidence of these mechanisms during our investigation of the *E. cuniculi* DNA repair proteome. Considering the extremely low levels of heterozygosity observed in *Encephalitozoon* species (Selman et al. 2013), LOH dynamics seem rather unlikely in Encephalitozoonidae.

Although the structural homology approach used in this study allowed us to identify several new components of the *E. cuniculi* DNA repair proteome, we were unable to detect all previously missing components, and we cannot rule out that other components might be left to be discovered for the following reasons. Not every protein structure could be predicted by template- and deep-learning-based tools, and of the predicted ones, some were somewhat discombobulated and likely erroneously folded (e.g., 89 [4.26%] and 366 [17.54%] of the protein structures predicted with AlphaFold averaged pLDDT scores smaller than 50% and 70%, respectively; supplementary table S1 and fig. S1, Supplementary Material online). Likewise, not all predicted structures had structural matches against experimental data from the RCSB PDB database, with 52.4% and 60.8% of the AlphaFold and RaptorX top-ranked models matching putative homologs at a *Q*-score cutoff of 0.3 (supplementary fig. S2, Supplementary Material online). Furthermore, structural homology by itself is insufficient to distinguish between highly repetitive folds, for example, the seven-bladed single β-propeller found in CSA, DDB2, and in so many more proteins (Henning et al. 1995; Fischer et al. 2011; Schapira et al. 2017), and the lack of sequence homology for many of the proteins featuring these repetitive folds prohibited us from assigning them with putative functions based solely on in silico inferences.

Nonetheless, considering the presence of the CSA-related components and a large number of possible structural analogs in the *E. cuniculi* proteome (supplementary table S3, Supplementary Material online), we hypothesize that CSA might indeed be present in this organism. Similarly, the presence of a DDB2 structural analog in the *E. cuniculi* proteome is also possible, but it is unclear if a DDB1–DDB2-like heterodimer should be expected in Microsporidia. In *Schizosaccharomyces pombe*, DDB1 was found to interact with several β-propeller-forming WD40 repeat proteins (Fukumoto et al. 2008) including the CSA homolog Ckn1 to protect DNA from UV damage. However, the budding yeast uses a DDB2-independent process facilitated by the Rad7–Rad16 complex to repair CPDs (Verhage et al. 1994), a complex for which we found no evidence in *E. cuniculi*. An impaired CPD lesion recognition would lead to an increased sensitivity to UV-damage (Fischer et al. 2011), a feature observed for *Encephalitozoon* spores (Marshall et al. 2003), and in vitro work will likely be required to properly assess the ability of this species to repair UV damage.

## Conclusion

The presence of a much more complete DNA repair proteome than previously anticipated in *E. cuniculi* and other microsporidians raises interesting questions about the evolutionary mechanisms that led to their genetic diversity. Whereas we can no longer assume that this diversity arose predominantly from a paucity of DNA repair proteins, we hypothesize that microsporidia (like many other unicellular pathogens including fungi) might use a hypermutator phenotype to adapt to the constraints of their obligate intracellular environments. Further biochemical studies will be required to test if the highly divergent DNA repair proteins in microsporidia are less effective at their task, thus enabling hypermutability. The present study was made possible with the latest developments in structural homology, and we expect this approach to become even more effective as more reference structures become available in databases. Albeit still somewhat computationally intensive, structural homology approaches are clearly becoming a strong complement to sequence homology tools for protein annotation.

## Materials and Methods

### Datasets

The *E. cuniculi* GB-M1 genome (Katinka et al. 2001), annotations, and protein dataset were downloaded from NCBI RefSeq (O’Leary et al. 2016) (accession GCF_000091225.1) and from MicrosporidiaDB (Aurrecoechea et al. 2011). Lists of GB-M1 proteins and their products were generated from the NCBI and MicrosporidiaDB GFF annotation files with get_GBM1_annotations.pl. *E. cuniculi* GB-M1 RNAseq datasets (Grisdale et al. 2013) at 24H (SRR769604, SRR769605), 48H (SRR769606, SRR769607), and 72H (SRR769608, SRR769609) postinfection were downloaded from the NCBI sequence read archive (SRA) (Leinonen et al. 2011) with fasterq-dump from the NCBI SRA Toolkit (v2.11.0; https://github.com/ncbi/sra-tools). Other Microsporidia protein datasets used in this study were downloaded from MicrosporidiaDB.

### Sequence Homology Searches

Pfam (Mistry et al. 2021) and CDD (Lu et al. 2020) searches were performed using InterProScan v5.51-85.0 (Jones et al. 2014). PSI-BLAST (Oda et al. 2017) homology searches were performed with up to three iterations against the NCBI nonredundant protein database. PSI-BLAST-directed searches against Microsporidia using human and yeast DNA repair protein orthologs were performed by restricting the search space to the microsporidian taxonomic ID (taxid:6029). Reversed HMM searches, that is, HMM models searched against sets of proteins, were performed using the MMH pipeline (https://github.com/PombertLab/MMH) with models built from protein datasets of representative microsporidia species (supplementary data S2, Supplementary Material online).

### Protein Structure Prediction

Protein structure predictions were performed on local workstations with the template-based RaptorX (Källberg et al. 2012) and the deep-learning-based AlphaFold2 (Jumper et al. 2021) (supplementary data S3, Supplementary Material online), as implemented in 3DFI v0.7a (Julian et al. 2021). RaptorX predictions (CNFpred 1.66; database v2019-02-28; Modeller v9.21, Webb and Sali 2016) were automated with raptorx.pl v0.6c from 3DFI. AlphaFold2 v2.0 predictions with the “–full_dbs” preset (databases v2021-07-19) were automated with alphafold.pl v0.3a from 3DFI with the “–max_template_date” option set to 2021-07-21 and computed using GPU acceleration on an NVIDIA RTX A6000 (Santa Clara, CA, USA). Average AlphaFold pLDDT scores were extracted from their corresponding ranking_debug.json files using get_pLDDTs.pl. Because the early AlphaFold2 version used did not produce PDB files with pLDDT per-residue confidence scores included, the per-residue pLDDT scores were extracted from the.pkl files and added to the b-factor column with the extract_b_values.py v0.2.8 and add_b_values.pl v0.2.8 Python and Perl scripts, respectively. A total of 28 *E. cuniculi* GB-M1 proteins could not be folded with the “–full_dbs” preset as they ran into a TensorFlow limitation, producing a “tensor proto > 2GB” error. These proteins were folded again using a newer version of AlphaFold2 (v2.1.1) and the “–reduced_dbs” preset instead (databases v2022-01-04) as implemented in 3DFI v0.9.0. Of these, two still ran over the 2GB TensorFlow limitation and could not be folded with AlphaFold2, whereas three ran partially resulting in a single unrelaxed model (supplementary table S4, Supplementary Material online). Protein complexes were reconstructed with AlphaFold-Multimer (Evans et al. 2021) from AlphaFold v2.1.1.

Per-residue confidence scores were further estimated independently using the deep convolutional neural network VoroCNN (Igashov et al. 2021), with per-protein average scores calculated with vorocnn_average.pl v0.3 on the proteins predicted by RaptorX and AlphaFold2 and on the reference RCSB PDB structures from tables 1 and 2 (supplementary data S4, Supplementary Material online). PDB files with VoroCNN per-residue scores in the b-factor columns were generated with color_pdb_vorocnn.pl v0.1a. Because RaptorX and AlphaFold2 did not yield high quality structures for the *T. hominis* DDB1, Cul4, and RBX1 proteins, these proteins were further folded independently with SWISS-MODEL (Waterhouse et al. 2018).

### Structural Homology Searches

The top-ranked AlphaFold and RaptorX models for each protein were searched for structural homologs against the experimentally determined structures from the RCSB PDB (Burley et al. 2021) with the General Efficient Structural Alignment of Macromolecular Targets (GESAMT) algorithm (Krissinel 2012) from the Collaborative Computational Project Number 4 (CCP4) package (Winn et al. 2011) (supplementary data S5, Supplementary Material online), as implemented in 3DFI v0.8a. Results were parsed with descriptive_GESAMT_matches.pl v0.7d to keep only the five best hits per model using a minimum *Q*-score of 0.1 (supplementary data S5, Supplementary Material online), and then further parsed with parse_all_models_by_Q.pl v0.1a, as implemented in 3DFI. Template modeling scores and associated root mean square deviation values were calculated for each of the structural matches with the MICAN-SQ algorithm from MICAN v2019-11-27 (Minami et al. 2018) using run_MICAN_on_GESAMT_results.pl. Global distance test scores were calculated with SPalign v2012-07-18 (Yang et al. 2012) using run_SPalign_on_GESAMT_results.pl. Round-robin structure comparisons between the per-protein AlphaFold predicted models (1–5) were performed with MICAN-SQ using compare_models_w_MICAN.pl. Predicted protein structures were visualized with ChimeraX v1.2.5 (Pettersen et al. 2021) and aligned against their putative structural homologs from the RCSB PDB database using ChimeraX’s built-in match function. To perform bidirectional searches, GESAMT archives were also generated from the protein structures predicted with RaptorX and AlphaFold2, and RCSB PDB reference structures of DNA repair proteins not identified in the previous genome-wide searches were queried against the RaptorX and AlphaFold2 GESAMT archives with run_GESAMT.pl v0.5e from 3DFI. Putative CSA and DDB2 homologs in the *E. cuniculi* proteome were inferred by performing GESAMT searches using the human CSA (6FCV chain B) and DDB2 (4A0A chain B) reference structures from RCSB PDB against the RaptorX and AlphaFold2 predicted protein structures. Gene ontologies were searched for in the 3D space with the COFACTOR program from the I-TASSER Suite 5 (Yang et al. 2015) package, using the *E. cuniculi* RaptorX structures as queries and the parallel_COFACTOR.pl v0.1b custom script (supplementary data S1, Supplementary Material online).

### Amino Acid Conservation and Electrostatic Potential

The human PCNA and Rad9–Rad1–Hus1 structures (accession numbers 3JA9 and 3A1J, respectively) were downloaded from the RCSB PDB database, and protein chains in the PDB files were separated into individual files using split_PDB.pl from 3DFI. Protein structures were aligned pairwise in the tridimensional space with GESAMT v1.16 using the human structures as query and the *E. cuniculi*/*T. hominis* proteins as target structures with run_gesamt_aln.pl. Pairwise identity and similarity percentages were calculated from the GESAMT alignments with 3D_align_stats.pl. Conserved amino acid residues were color-coded with ChimeraX using the default AL2CO (Pei and Grishin 2001) entropy-based method from the “color byattribute seq_conservation” command. Surface electrostatic potentials were calculated with ChimeraX using the command “coulombic protein range −8,8.”

### Gene Expression

The *E. cuniculi* RNAseq data at 24H, 48H, and 72H postinfection (Grisdale et al. 2013) were mapped against the *E. cuniculi* GB-M1 reference genome (assembly ASM9122v1) with minimap2 v2.17 (Li 2018) with the short read (sr) preset in paired-end mode using get_SNPs.pl v2.0 from the SSRG pipeline (https://github.com/PombertLab/SSRG). Expression levels for each of the CDS, tRNA, and RNA genes present in the *E. cuniculi* GB-M1 NCBI GFF annotation file (supplementary data S6, Supplementary Material online) were estimated with genes_expressed.pl v0.5 from the sequencing depth at each position listed in the *.coverage files generated by get_SNPs.pl and derived from the SAMtools v1.11 “depth -aa” function (Li et al. 2009).

### Phylogenetic Tree

Phylogenetic relationships between microsporidia species represented in figures 1 and 3 were inferred from an alpha-tubulin maximum likelihood (ML) tree as follows. Alpha-tubulin sequences were identified in the downloaded protein datasets by BLASTP sequence homology using the *E. intestinalis* tubulin sequence as query (accession number XP_003073238.1). Tubulin protein sequences were aligned with Clustal Omega v1.2.4 (Sievers et al. 2011). The best ML tree was inferred with PhyML v3.1 (Guindon et al. 2010) using an initial BioNJ tree, the LG model of amino acid substitutions, and four gamma categories. The tree generated (in nexus format) was converted to a cladogram with FigTree v1.4.4 (http://tree.bio.ed.ac.uk/software/figtree/) using *Mitosporidium daphniae* as outgroup.

## Supplementary Material

evac053_Supplementary_Data

## Data Availability

ChimeraX alignment files are available in the supplementary data S7, Supplementary Material online. The datasets generated in this study (supplementary data S1–S7, Supplementary Material online) are publicly available on Zenodo (DOI: 10.5281/zenodo.6360725).

## Code Availability

All custom scripts and related files used in this study are available online on GitHub (https://github.com/PombertLab) and on Zenodo (DOI: 10.5281/zenodo.6360725) as supplementary data S8, Supplementary Material online.
