# Supplementary material for: The Rad9–Rad1–Hus1 DNA Repair Clamp is Found in Microsporidia
Source: Genome Biol Evol. 2022 Apr 19;14(4):evac053. doi: 10.1093/gbe/evac053 (PMC9053307; doi:10.1093/gbe/evac053)
Supplement: evac053_Supplementary_Data [file evac053_supplementary_data.zip › Supplementary information.docx]

**Supplementary information**

**Supplementary Figure 1. Distribution of the pLDDT and voroCNN folding metrics for the predicted *E. cuniculi* GB-M1 proteins.** *(A)* pLDDT score distribution for the AlphaFold predicted structures. *(B)* voroCNN score distribution for the AlphaFold and RaptorX structures. (*A* *and* *B*) Proteins with putative functions in the MicrosporidiaDB annotations are represented in orange in the stacked histograms; hypothetical proteins are represented in blue. Hypothetical proteins include proteins annotated as hypothetical, uncharacterized and UPF (Unknown Protein Function). *(C)* Scatter plot comparison of the AlphaFold pLDDT scores (x-axis) and corresponding voroCNN scores (y-axis). The fitted linear relationship is plotted as a black line; the non-fitted 1-to-1 linear relationship is plotted as an orange line for visual comparison. *(A to C)* Data for these plots is available in Supplementary Table 1.

**Supplementary Figure 2. Distribution of the Q-score and TM-score structural homology metrics for the predicted *E. cuniculi* GB-M1 proteins.** *(A)* Q-score distribution for the AlphaFold/Raptorx predicted structures and their top ranked structural homolog (Q-score ≥ 0.1). *(B)* TM-score distribution for the AlphaFold/RaptorX and their top ranked structural homolog. (*A* *and* *B*) Proteins with putative functions in the MicrosporidiaDB annotations are represented in orange in the stacked histograms; hypothetical proteins are represented in blue. Hypothetical proteins include proteins annotated as hypothetical, uncharacterized and UPF (Unknown Protein Function). *(C)* Scatter plot comparison of the AlphaFold Q-scores (x-axis) and corresponding TM-scores (y-axis). Possible fitted linear/quadratic relationships are plotted as black and blue lines, respectively; the non-fitted 1-to-1 linear relationship is plotted as an orange line for visual comparison. Only matches with Q-scores ≥ 0.1 were considered as potential structural homologs. *(A to C)* Data for these plots is available in Supplementary Table 1.

**Supplementary Figure 3. VoroCNN quality assessment of experimentally determined and computationally predicted Rad9, Rad1, Hus1 protein structures**. *Top*. VoroCNN quality assessment of the crystal structure of the human 9-1-1 complex (RCSB PDB structure 3A1J). *Center*. VoroCNN quality assessment of the *E. cuniculi* 9-1-1 complex predicted with RaptorX. *Bottom*. VoroCNN quality assessment of the *E. cuniculi* 9-1-1 complex predicted with AlphaFold2.

**Supplementary Figure 4. PCNA/911 protein complexes as predicted by AlphaFold-Multimer.** *Panels A and B.* Predicted structure of the homotrimeric PCNA clamp (3x ECU05_1030) colored by chain (A) and by pLDDT confidence scores (B). *Panels C and D.* Predicted structure of the heterotrimeric 911 clamp (ECU07_1290, ECU08_0130, and ECU08_0200) colored by chain (C) and by pLDDT confidence scores (D). In panel C, ECU07_1290, ECU08_0130 and ECU08_0200 are colored in blue, red and green, respectively.

**Supplementary Figure 5. Conserved amino acids between the *E. cuniculi*/*T. hominis* PCNA-like structures and their human counterparts.** Conserved residues were inferred from structure alignments of the microsporidian proteins against the human experimental structures with GESAMT and color-coded with AL2CO as implemented in ChimeraX.

**Supplementary Figure 6. Examples of DDB1-Cul4-RBX1 protein complexes as predicted by AlphaFold-Multimer.** *Panels A and B.* Predicted structure of DDB1-Cul4-RBX1 (ECU05_1150 -ECU06_0880 -ECU01_1095) colored by chain (A) and by pLDDT confidence scores (B). *Panels C and D.* Predicted structure of DDB1-Cul4-RBX1 (ECU11_0610-ECU06_0880 -ECU01_1095) colored by chain (C) and by pLDDT confidence scores (D). In panels A and C, DDB1, Cul4 and RBX1 are colored in green, red and blue, respectively.
