# Supplementary material for: The Rad9–Rad1–Hus1 DNA Repair Clamp is Found in Microsporidia
Source: Genome Biol Evol. 2022 Apr 19;14(4):evac053. doi: 10.1093/gbe/evac053 (PMC9053307; doi:10.1093/gbe/evac053)
Supplement: evac053_Supplementary_Data [file evac053_supplementary_data.zip › Supplementary_Figures.pdf]

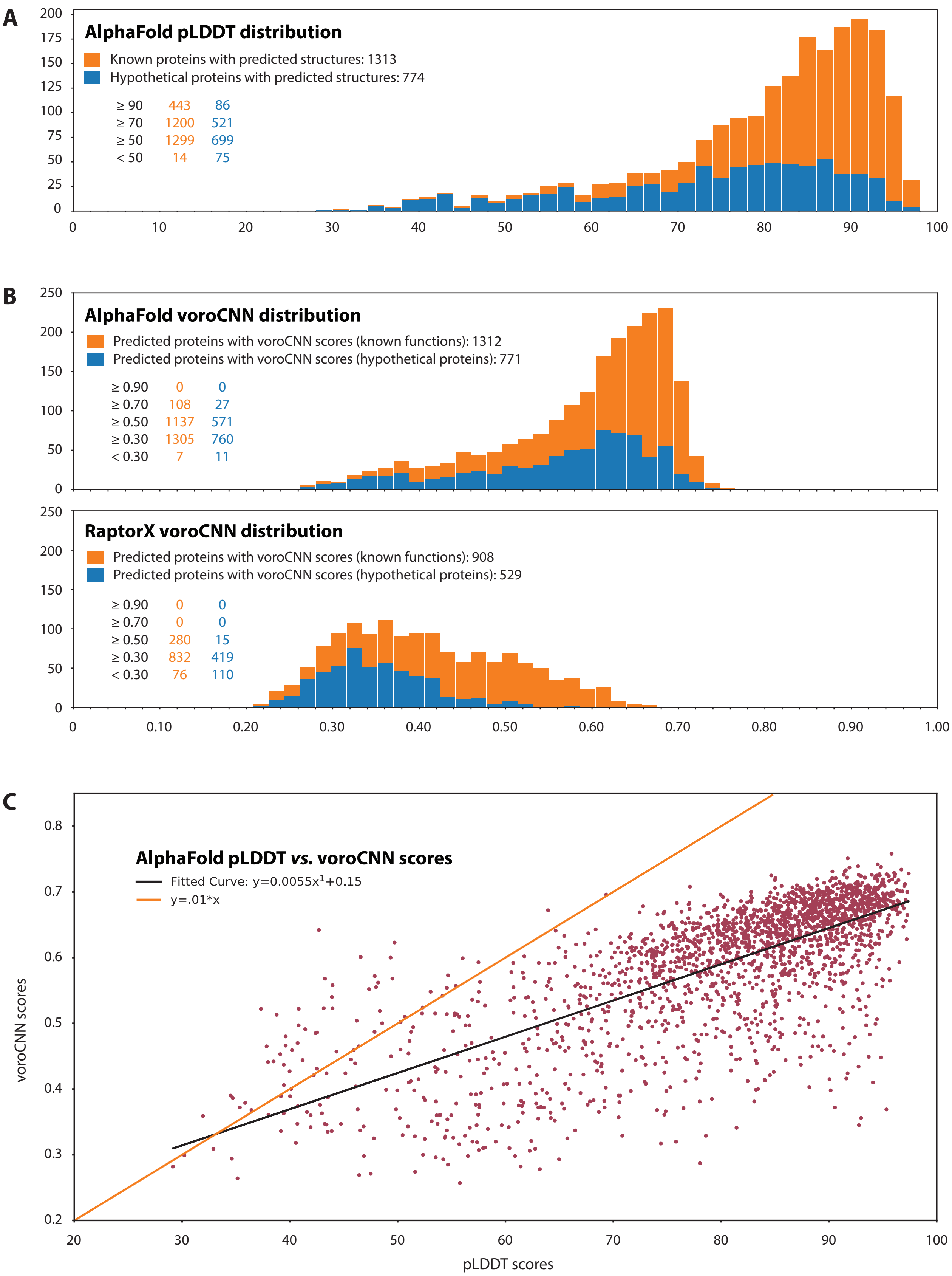

Supplementary Figure 1

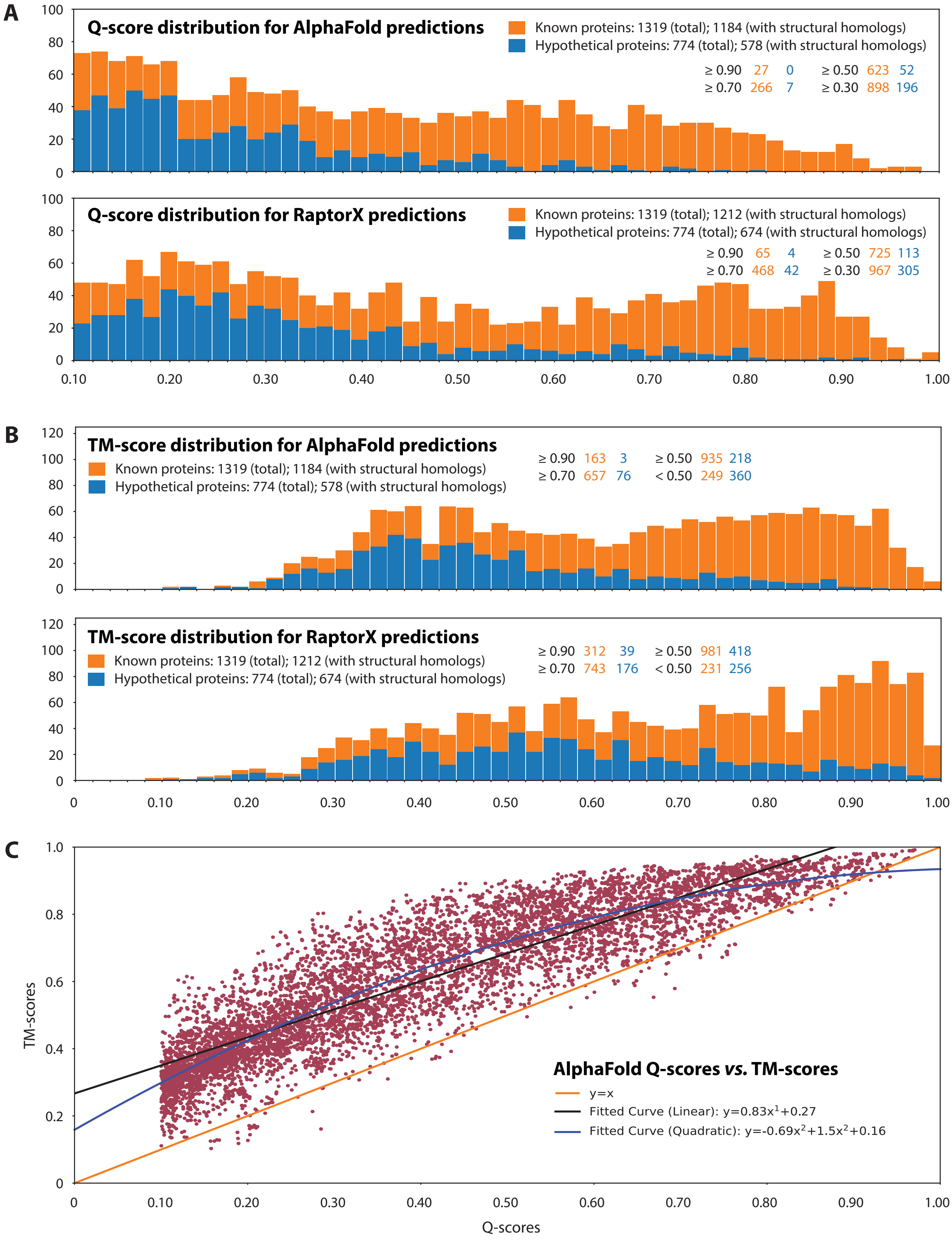

Supplementary Figure 2

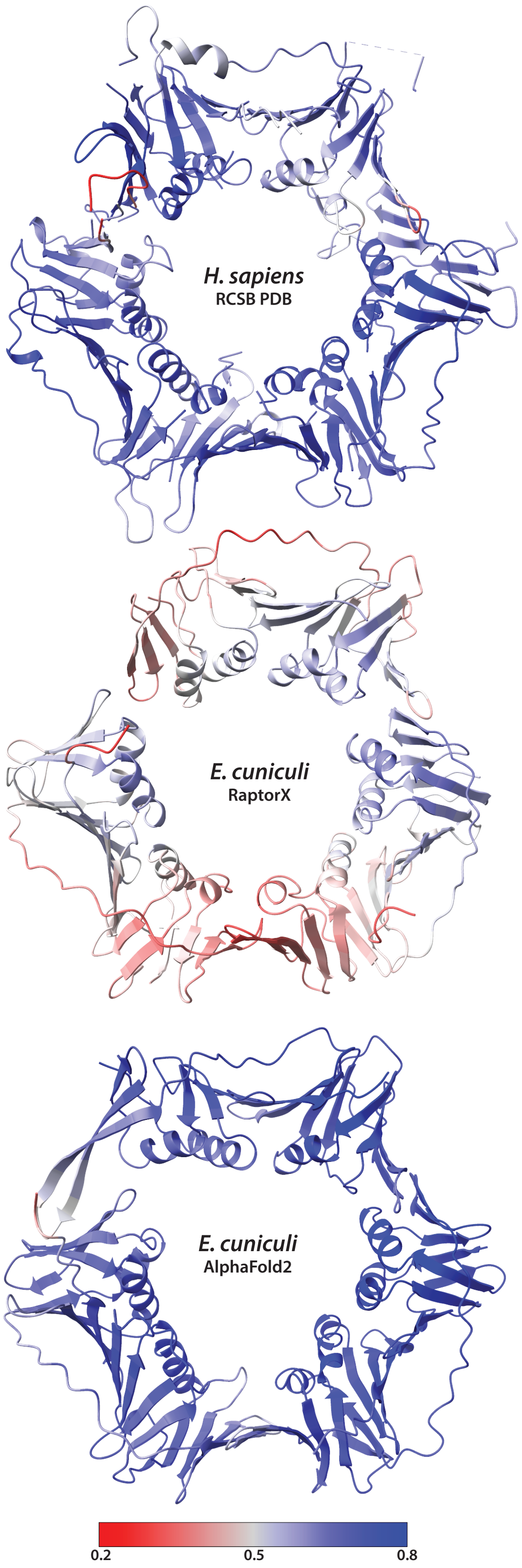

Supplementary Figure 3

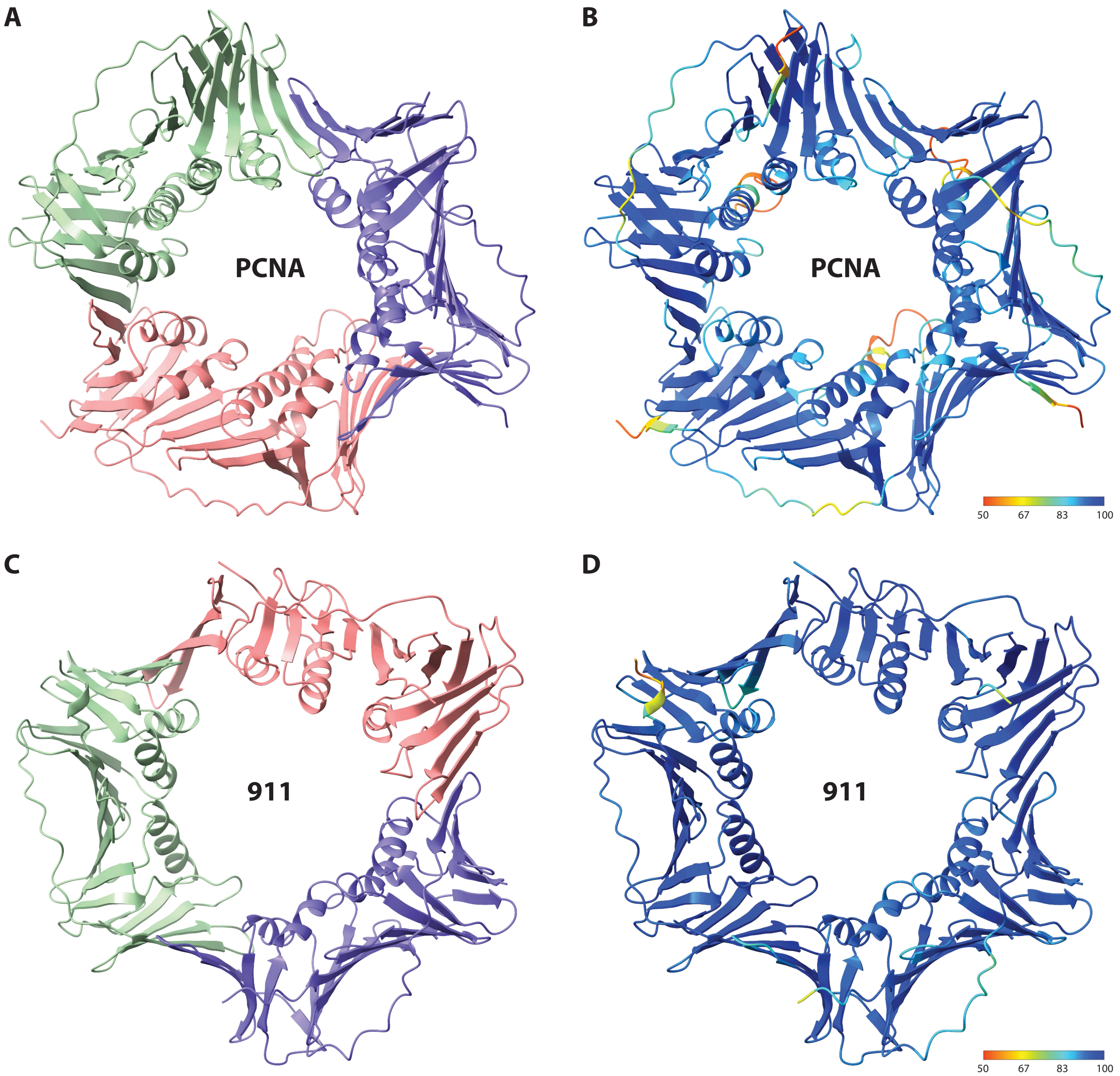

Supplementary Figure 4

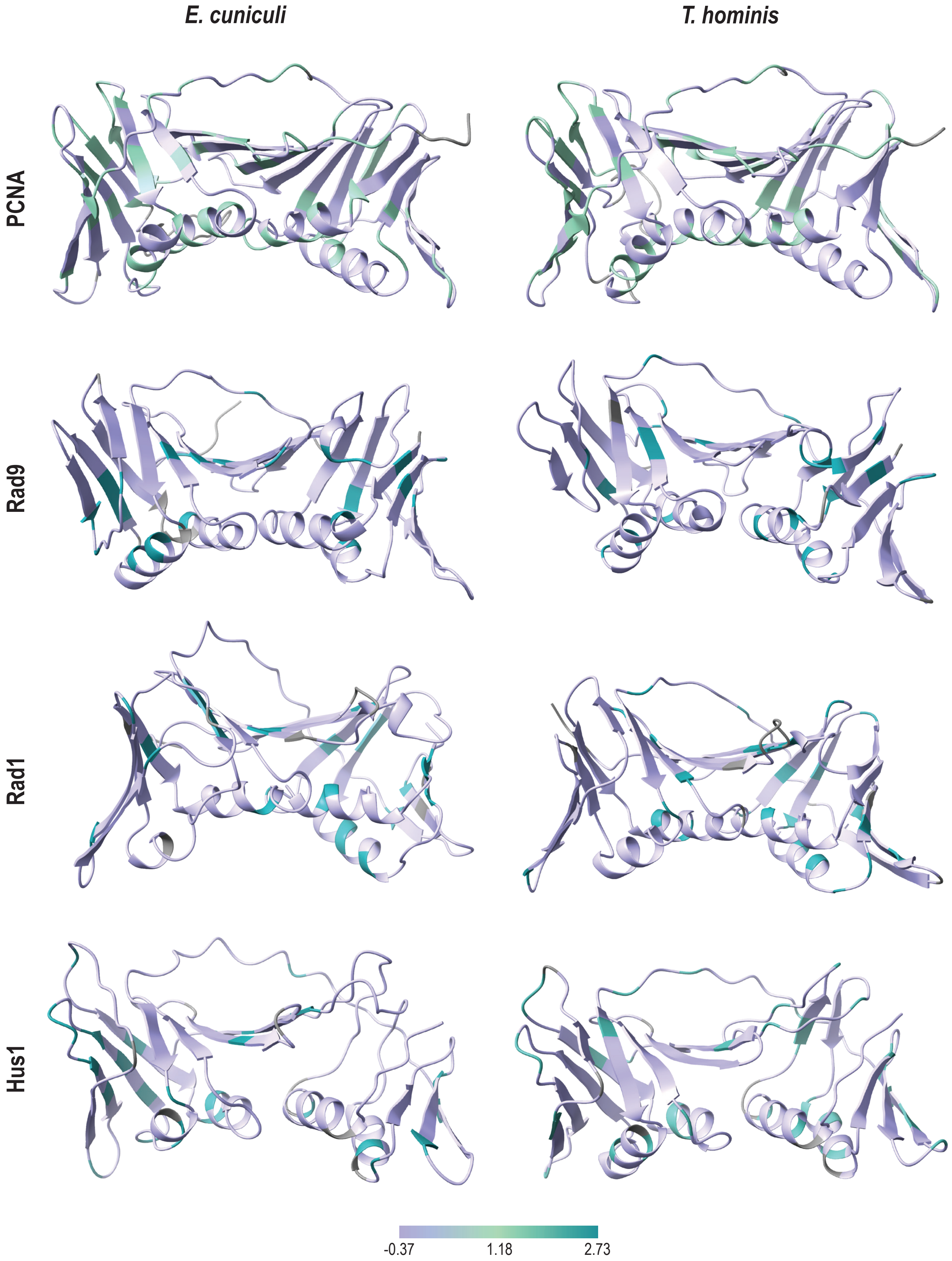

Supplementary Figure 5

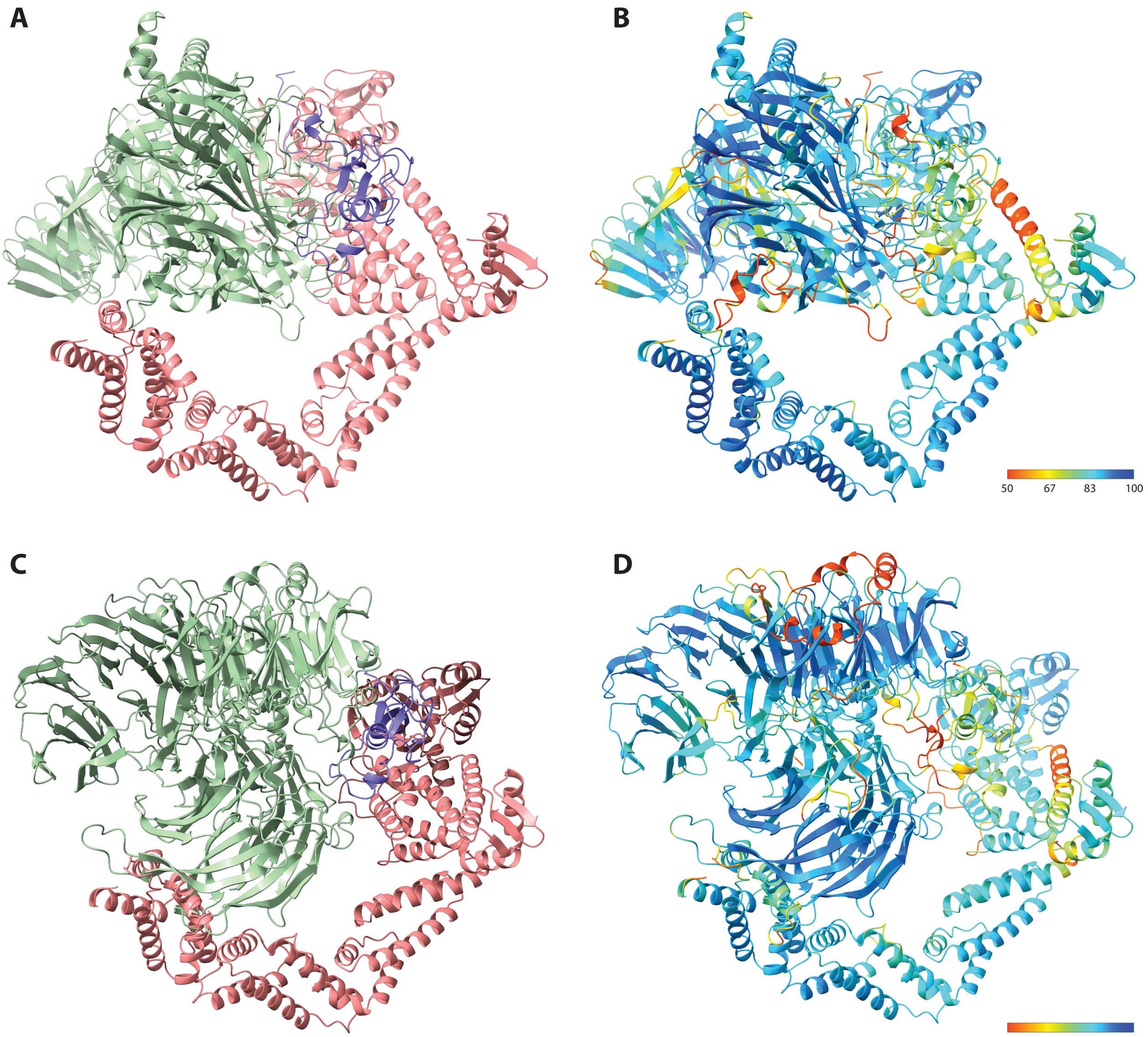

Supplementary Figure 6
