# Supplementary material for: The Rad9–Rad1–Hus1 DNA Repair Clamp is Found in Microsporidia
Source: Genome Biol Evol. 2022 Apr 19;14(4):evac053. doi: 10.1093/gbe/evac053 (PMC9053307; doi:10.1093/gbe/evac053)
Supplement: evac053_Supplementary_Data [file evac053_supplementary_data.zip › Supplementary_Table_3.docx]

| **Supplementary Table 3.** Putative CSA and DDB2 structural analogs in *E. cuniculi* GB-M1^*^. | | | | |  |
| --- | --- | --- | --- | --- | --- |
|  |  |  | **Q-score against reference structure^b^** | | |
| **Locus tag** | **Putative function (from accession number^a^)** | **Length(aa)** | **CSA** | **DDB2** |  |
| ECU02_0240 | U3 snoRNA-associated RNP (18s RRNA production) | 408 | 0.3506 | 0.3337 |  |
| ECU02_1400 | similarity to hypothetical WD-repeat protein YN57_yeast | 408 | 0.4306 | 0.3753 |  |
| ECU03_1110 | hypothetical protein | 277 | 0.3813 | 0.3645 |  |
| ECU03_1230 | similarity to periodic tryptophan protein 1 | 330 | 0.3535 | 0.3559 |  |
| ECU03_1520 | similarity to CDC20 (WD-repeat protein) | 369 | 0.3821 | 0.358 |  |
| ECU04_1250 | CDC20-like protein | 362 | 0.4125 | 0.3893 |  |
| ECU04_1320 | hypothetical protein | 252 | --- | 0.3083 |  |
| ECU04_1580 | similarity to hypothetical protein YKB8_yeast | 294 | 0.4261 | 0.4375 |  |
| ECU05_0550 | hypothetical protein | 350 | 0.3334 | 0.3115 |  |
| ECU05_0810 | ser/thr protein phosphatase PP2-A regulatory subunit B | 368 | 0.3752 | 0.3446 |  |
| ECU05_1010 | beta-transducin repeat containing protein | 338 | 0.4027 | 0.3762 |  |
| ECU05_1500 | similarity to hypothetical WD-repeat protein YER2_yeast | 435 | 0.3215 | 0.3134 |  |
| ECU06_0970 | hypothetical protein | 298 | 0.3355 | --- |  |
| ECU06_1550 | mRNA associated protein of the RAE1 family | 318 | 0.4322 | 0.3193 |  |
| ECU07_0150 | hypothetical protein | 294 | 0.4005 | 0.3768 |  |
| ECU07_0280 | hypothetical protein | 280 | 0.4626 | 0.4379 |  |
| ECU07_0750 | histone acetyltransferase type B subunit 2 | 384 | 0.4255 | 0.3739 |  |
| ECU07_1020 | putative WD-repeat protein | 401 | 0.376 | 0.3396 |  |
| ECU08_0310 | similarity to guanine nucleotide binding protein beta subunit gblp_drome | 298 | 0.5011 | 0.4405 |  |
| ECU08_0690 | poly(A)+ RNA export protein | 287 | 0.4284 | 0.3884 |  |
| ECU08_1110 | guanine nucleotide binding protein beta subunit | 334 | 0.51 | 0.4306 |  |
| ECU08_1240 | WD repeat containing protein | 296 | 0.3643 | 0.36 |  |
| ECU08_1260 | chromatin assembly factor 1 P60 subunit | 364 | 0.376 | 0.3439 |  |
| ECU09_1480 | hypothetical protein | 280 | 0.502 | 0.4586 |  |
| ECU09_1660 | hypothetical protein | 319 | 0.4195 | 0.342 |  |
| ECU09_1770 | guanine nucleotide-binding protein beta subunit | 312 | 0.4319 | 0.3902 |  |
| ECU10_0220 | translation initiation factor IF3 subunit 2 | 329 | 0.4555 | 0.4209 |  |
| ECU11_1450 | protein transport protein SEC13 homolog (COPII coat) | 272 | 0.4068 | 0.3657 |  |
| ECU11_1970 | hypothetical protein | 280 | 0.5554 | 0.4766 |  |

^a^ NCBI RefSeq accession number GCF_000091225.1

^b^ RCSB PDB reference structures for CSA and DDB2 are 6FCV chain B and 4A0A chain B, respectively.

^*^ This table was generated with the custom Perl script gesamt_table.pl (see Supplementary Data 8).
