# Supplementary material for: The Rad9–Rad1–Hus1 DNA Repair Clamp is Found in Microsporidia
Source: Genome Biol Evol. 2022 Apr 19;14(4):evac053. doi: 10.1093/gbe/evac053 (PMC9053307; doi:10.1093/gbe/evac053)
Supplement: evac053_Supplementary_Data [file evac053_supplementary_data.zip › Supplementary_Table_4.docx]

**Supplementary Table 4.** Proteins that could not be folded with AlphaFold's '--full_dbs' preset.

| **Locus tag** | **Predicted function** | **Length (aa)** | |
| --- | --- | --- | --- |
| ***Folded with --reduced_dbs*** | | |  |
| ECU03_0240 | ABC transporter | 585 | |
| ECU03_0390 | ABC transporter | 590 | |
| ECU04_0320 | hypothetical protein | 607 | |
| ECU04_0910 | putative ATP-dependent RNA helicase (SKI2 subfamily) | 933 | |
| ECU04_1290 | ATP-dependent RNA helicase (SKI2 family) | 881 | |
| ECU05_0100 | hypothetical protein | 153 | |
| ECU05_0310 | acetylcoenzyme A synthetase | 632 | |
| ECU06_0920 | pre-mRNA splicing helicase | 1481 | |
| ECU07_1060 | ATP-dependent RNA helicase (DEAD box family) | 416 | |
| ECU07_1130 | ATP-dependent DNA helicase | 766 | |
| ECU08_0110 | probable ABC transporter | 669 | |
| ECU08_2050 | hypothetical protein | 466 | |
| ECU09_0090 | hypothetical protein | 1137 | |
| ECU09_0180 | hypothetical protein | 489 | |
| ECU10_0700 | translation initiation factor EIF-2B epsilon subunit | 569 | |
| ECU10_0890 | long chain fatty acid CoA ligase | 626 | |
| ECU10_0910 | long chain fatty acid CoA ligase | 708 | |
| ECU10_1230 | ABC transporter | 572 | |
| ECU10_1520 | ABC transporter | 678 | |
| ECU11_0490 | hypothetical protein | 537 | |
| ECU11_1200 | ABC transporter (mitochondrial type) | 600 | |
| ECU11_1420 | HSP 101 related protein | 851 | |
| ECU11_1440 | hypothetical protein | 1303 | |
| ***Single model produced ^a^*** | | |  |
| ECU05_0540 | similarity to hypothetical protein of the PI3/PI4 kinase family | 3436 | |
| ECU08_1900 | putative protein of the CBBQ/NORQ/Ncd IRQ/GVPN family | 2832 | |
| ECU10_0640 | dynein heavy chain | 3151 | |
| ***Could not be folded ^b^*** | | |  |
| ECU01_0200 | ABC transporter | 596 | |
| ECU01_1410 | ABC transporter | 596 | |

^a^ Proteins for which a single unrelaxed model was produced with --reduced_dbs before the computation crashed.

^b^ Proteins that could not be folded with --full_dbs or --reduced_dbs due to AlphaFold2 running into a TensorFlow limitation, producing a 'tensor proto > 2GB' error.
